# Cognitive Dysfunction, Brain Volumes, and Traumatic Brain Injury in Homeless Persons

**DOI:** 10.1089/neur.2020.0031

**Published:** 2021-03-05

**Authors:** Michael D. Cusimano, Ashirbani Saha, Daniel Zhang, Stanley Zhang, Julia Casey, Jessica Rabski, Melissa Carpino, Stephen W. Hwang

**Affiliations:** ^1^ Injury Prevention Research Office, Division of Neurosurgery, Li Ka Shing Knowledge Institute, St. Michael's Hospital, Toronto, Ontario, Canada.; ^2^ Dalla Lana School of Public Health, University of Toronto, Toronto, Ontario, Canada.; ^3^ Department of Medicine, University of Toronto, Toronto, Ontario, Canada.; ^4^ Centre for Urban Health Solutions, St. Michael's Hospital, Toronto, Ontario, Canada.

**Keywords:** biomarkers, brain volume, cognitive function, concussion, homeless persons, neuroimaging, random forest, traumatic brain injury

## Abstract

Although homeless persons experience traumatic brain injury (TBI) frequently, little is known about the structural and functional brain changes in this group. We aimed to describe brain volume changes and related cognitive/motor deficits in homeless persons with or without TBI versus controls. Participants underwent T1-weighted magnetic resonance imaging (MRI), neuropsychological (NP) tests (the Grooved Pegboard Test [GPT]/Finger Tapping Test [FTT]), alcohol/drug use screens (the Alcohol Use Disorders Identification Test [AUDIT]/Drug Abuse Screening Test [DAST]), and questionnaires (the Brain Injury Screening Questionnaire [BISQ]/General Information Questionnaire [GIQ]) to determine TBI. Normalized volumes of brain substructures from MRI were derived from FreeSurfer. Comparisons were tested by Mann-Whitney U and Kruskal-Wallis rank sum tests. Leave-one-out cross-validation using random forest classifier was applied to determine the ability of predicting TBI. Diagnostic ability of this classifier was assessed using area under the receiver operating characteristic curve (AUC). Fifty-one participants—25 homeless persons (9 with TBI) and 26 controls—were included. The homeless group had higher AUDIT scores and smaller thalamus and brainstem volumes (*p <* 0.001) than controls. Within homeless participants, the TBI group had reduced normalized volumes of nucleus accumbens, thalamus, ventral diencephalon, and brainstem compared with the non-TBI group (*p <* 0.001). Homeless participants took more time on the GPT compared with controls using both hands (*p <* 0.0001); but the observed effects were more pronounced in the homeless group with TBI in the non-dominant hand. Homeless persons with TBI had fewer dominant hand finger taps than controls (*p =* 0.0096), and homeless participants with (*p =* 0.0148) or without TBI (*p =* 0.0093) tapped less than controls with their non-dominant hand. In all participants, TBI was predicted with an AUC of 0.95 (95% confidence interval [CI]: 0.89-1.00) by the classifier modeled on MRI, NP tests, and screening data combined. The MRI-data-based classifier was the best predictor of TBI within the homeless group (AUC: 0.76, 95% CI: 0.53-0.99). Normalized volumes of specific brain substructures were important indicators of TBI in homeless participants and they are important indicators of TBI in the state of homelessness itself. They may improve predictive ability of NP and screening tests in determining these outcomes.

## Introduction

Traumatic brain injury (TBI) is one of the most common neurological conditions. The Centers for Disease Control and Prevention defines TBI as any injury to the head that disrupts homeostatic functioning of the brain.^1^ The importance of TBI should not be underestimated, as it is responsible for more trauma-related deaths than injury to any other region of the body.^1^ TBI has been reported as a major cause of death and disability in the United States and accounts for 30% of all injury deaths.^2^ The annual incidence rate of TBI surpasses the annual incidence of breast cancer, HIV/AIDS, spinal cord injuries, and multiple sclerosis combined.^1^

People experiencing homelessness are disproportionately affected by TBI. Evidence suggests that TBI is two to seven times more prevalent in the homeless population compared with the general public.^3–6^ This finding is consistent across multiple metropolitan areas, each with significantly different general and homeless population sizes. A recent meta-analysis showed that the lifetime prevalence of any severity of TBI in homeless and marginally housed individuals was 53.1% (95% confidence interval [CI]: 46.4-59.7) and the lifetime prevalence of moderate or severe TBI was 22.5% (95% CI: 13.5-35.0).^7^ Homeless individuals with TBI are more likely to live with chronic health conditions, experience poorer physical and mental health, and suffer from drug and alcohol abuse compared with homeless persons without TBI.^3,6^ Further, TBI in the homeless population has been shown to be an independent risk factor of increased probability of arrest or incarceration, chance of being physically assaulted, number of emergency department visits, and mortality.^4,6^ Despite the negative consequences of high TBI prevalence among homeless individuals, it remains underdiagnosed and inappropriately treated in this population.^8,9^

Previous research has indicated that motor deficits resulting from TBI are correlated with cognitive impairments, the combination of which may interfere with an individual's ability to benefit from treatment services.^9,10^ The Finger Tapping Test (FTT) has been validated widely to measure the performance speed of a simple motor task and has demonstrated approximately 10% higher motor speed for dominant hand performance when compared with non-dominant hand performance.^11,12^ The mean speed of finger tapping has subsequently also been shown to correlate with severity of TBI.^13^ Meanwhile, the Grooved Pegboard Test (GPT) was developed to assess fine motor speed, which requires both manual dexterity and complex motor coordination.^11,14^ Some studies have suggested its use as a test of cognitive function, in addition to its motor components, as a result of its ability to reflect the cognitive decline associated with postural instability and falls in patients with Parkinson's disease.^15^ Although a significant amount of literature exists pertaining to the use of both of these neuropsychological (NP) tests in the general population,^14,16–18^ the literature regarding their measurement in homeless populations remains scarce.

Although research examining the use of magnetic resonance imaging (MRI) to investigate neuroanatomical characteristics is increasingly prevalent in the TBI population, such studies in the homeless population remain limited. For instance, a small study investigating homeless, crack-cocaine-dependent African-American men (*n* = 9) in comparison with healthy controls (*n* = 8), used voxel based morphometry and a region of interest (ROI) analysis and showed that homeless cocaine-dependent individuals had smaller gray matter volume in the dorsolateral prefrontal cortex, anterior cingulate, cerebellum, insula, and superior temporal gyrus.^19^ The small sample size and coexistent addiction prevented the authors from commenting on the individual effects of addiction and homelessness, and there were no measures of cognitive function reported. Another study investigated the volume of the amygdala and the hippocampus in underhoused individuals and found that individuals with the larger amygdala and central nucleus volumes had a larger social network size.^20^ This study did not comment on TBI or on cognitive functions of the individuals.^20^ As such, critical gaps remain in explicating the relationship between neuroanatomical findings and NP function in homeless populations.

Alcohol and drug abuse continue to represent a major public health problem among the homeless population, and plays a significant role in their higher rates of victimization and arrests.^21^ Conservative estimates state the prevalence of such abuse to be roughly 50% within the homeless population. Due to this high prevalence of alcohol and drug abuse and the strong association of recurrent and chronic homelessness with alcohol and drug abuse, we used the Alcohol Use Disorders Identification Test (AUDIT) and Drug Abuse Screening Test (DAST) to collect data on alcohol and drug abuse history for all study participants.

Factoring in these unique characteristics of homeless populations, the purpose of this study was to examine the relationship between neuroanatomical subsegmentation volumes obtained from T1-weighted MRI images, NP test performance (using the FTT and GPT), and alcohol or drug abuse history (using AUDIT and DAST scores) among homeless individuals with or without TBI. We hypothesize that homeless individuals will perform worse than non-homeless control groups on both the FTT and GPT, have higher AUDIT and DAST scores, and that their neuroanatomical subsegmentation volumes will be reduced, particularly in those who have experienced a previous TBI. Additionally, we tested the ability of NP tests, AUDIT scores, DAST scores, and subsegmental neuroanatomical volumes to predict the lifetime exposure of TBI in homeless participants.

## Methods

### Study design and ethics board approval

The study and consent procedure were approved by the Research Ethics Board of St. Michael's Hospital, Toronto, Ontario. Data collection was conducted prospectively at two different appointments, beginning after written informed consent was obtained for each participant. The homeless shelter team was given a presentation about the study and inclusion criteria for eligible participants. Eligible individuals with capacity to consent were briefly introduced to the study by a member of their direct circle of care. Research assistants were trained with all study procedures, including informed consent. If the consenting capacity of any participants was doubtful, they was excluded. The first appointment consisted of completing the General Information Questionnaire (GIQ) and the Brain Injury Screening Questionnaire (BISQ),^22,23^ and undergoing an NP assessment (using the FTT and GPT). A brain MRI scan was performed at the subsequent appointment.

### Recruitment and data collection

Homelessness, in this study, was defined as sleeping overnight at a homeless shelter, on the street, in a public place, or any other location not intended for human habitation. Homeless participants were recruited from meal programs and their associated homeless shelters situated in downtown Toronto, Ontario. Study participants in this group were 29–67 years old with or without a history of TBI. If they had sustained a TBI, inclusion into the study was permitted if their most recent TBI was no more than 3 months prior to study enrollment to ensure that participants were medically stable. Participants were excluded if they did not have adequate verbal English language skills, because all study questionnaires and NP assessments were conducted in English. Participants were also excluded if they were noticeably intoxicated at the time of recruitment, unable to undergo MRI scanning, medically unstable or required hospitalization, or if they presented with neurodegenerative disorders including severe clinical comorbidities such as multiple sclerosis, dementia, Parkinson's disease, or uncontrolled diabetes. A history of drug or alcohol abuse was not considered as a basis for exclusion unless this abuse manifested in neurodegenerative disorders.

Control group participants were recruited from the general public via study advertisement or referral approximately 1 year after all homeless participants were recruited. Controls were matched to homeless participants based on age, sex, and education.

### Questionnaires

The GIQ was used to obtain information regarding demographics, general health, neighborhood, conflict, leisure activities, and self-reported past history of TBI. The BISQ^22,23^ was used to determine the likelihood of lifetime TBI exposure by grouping participants into four different risk categories. The probability of TBI exposure determined by the BISQ was scored from 0 to 3, where 0 indicated no probability (henceforth referred to as “negative”), 1 low, 2 moderate, and 3 high probability of TBI exposure. BISQ scores were used in comparison with controls for NP testing results and MRI neuroanatomical subsegmental volumes. The DAST^24,25^ was used to determine whether participants were currently abusing or had abused drugs (excluding alcohol) in the past. DAST scores can range from 0 to 20 and can be divided into categories as follows: a score of 0 (no drug abuse), 1–5 (mild drug abuse), 6–10 (moderate drug abuse), 11–15 (substantial drug abuse), and 16–20 (severe drug abuse). The AUDIT^26^ is a 10-item screen for excessive drinking and hazardous alcohol use, with scores ranging from 0 to 40. Scores of 16 and above indicate problematic drinking, and scores of 20 and above indicate alcohol dependence.

### NP tests

In the FTT of manual motor speed to detect motor impairment,^27^ each participant was instructed to tap a mechanical tracker as rapidly as possible for 10 sec using their index finger. Five trials were conducted for each hand, starting with the participant's dominant hand. If the difference between the fastest and the slowest trial was greater than 5, an extra trial would be given so that five consecutive trials were attained within a 5-point range of each other. A maximum of 10 trials with each hand was allowed. The final score was calculated as the mean of the five trials for each hand separately, which diminishes the influence of a single deviant score on total performance.

The GPT assesses numerous cognitive-motor functions, such as fine motor speed, manual dexterity, and complex limb coordination. It has been shown to be a valid assessment of motor impairment, and is commonly included in NP batteries.^28^ The test requires participants to insert 25 irregularly-shaped pegs into their respective holes as quickly as possible using either their dominant or non-dominant hand. Scores for each hand are based on the time taken to complete the task. Time to task completion and number of accidental peg drops have been shown to correlate with age. As such, participant age in the homeless group was matched to that of the controls, allowing motor impairment to be discerned by scores that are significantly higher than normal for the participant's age and sex. A trial was stopped if the participant gave up on the task after expending significant effort, or if the participant failed to complete the task within 10 min. All FTT and GPT scores were converted to T-scores based on age, sex, education, and hand used, and based on the mean and standard deviation reported on normative data in the study.^29^

### Brain MRIs

Structural MRIs were conducted on a 3.0 Tesla system at Sunnybrook Health Sciences Centre (MR750, GE Healthcare, Waukesha, WI, USA), with a slew rate of 200 T/m/sec, maximum strength of the gradient 50 mT/m, with a 12-channel, phased-array head coil. The following procedures were completed for each participant: a three-dimensional, longitudinal relaxation time, T1-weighted sequence (slice thickness = 1.4 mm; flip angle = 15 degrees; repetition time [TR] = 8.2 msec; echo time [TE] = 3.2 msec; imaging—in-plane resolution = 0.9 mm × 0.9 mm; field of view [FOV] = 220 mm × 165 mm) to rule out gross structural abnormalities; a T2-weighted sequence; diffusion tensor imaging (DTI); fluid-attenuated inversion recovery (FLAIR); and gradient echo. The parameters for these MRI sequences followed previous study designs.^30^

Brain volumes were measured using FreeSurfer version 5.3.0 using the T1-weighted sequence.^31–33^ The volume-based stream was used, which consists of six stages: affine registration with the Montreal Neurological Institute Atlas (MNI305), initial volumetric labeling, B1 bias field correction, skull stripping, high-dimensional non-linear volumetric alignment to the MNI305 Atlas, and eventually data labeling (segmentation). A sum-total of 24 volumetric regions were calculated. The volumetric data were normalized using the proportion method^34^ by dividing the ROI volume by estimated total intracranial volume (ICV) computed using FreeSurfer. The volumes of the putamen, accumbens, ventral diencephalon, pallidum, and thalamus regions were selected after a neurosurgeon, blinded to the outcomes, visually confirmed the consistency of the segmentation of these regions.

### Statistical analysis

Although all participants completed the GIQ, missing data were recorded with the NP tests as follows: FTT dominant hand (*n* = 4), FTT non-dominant hand (*n* = 3), GPT dominant hand (*n* = 5), GPT non-dominant hand (*n* = 5), and AUDIT (*n* = 1). When using data for a specific test, only the participants with full corresponding data were considered. Two sets of analyses were performed for each of the NP tests and for the MRI brain volumes.

AUDIT scores, DAST scores, FTT scores, GPT scores, and brain volumes were compared between the homeless and control groups. These intergroup comparisons were conducted using the Mann-Whitney U test (chosen when using non-parametric and independent variables that are continuous and ordinal). A level of significance 0.0042 (0.05/12) was used to account for multiple hypothesis testing in our work. Data that achieved less than a 0.0042 level of significance are marked by ** to separate those from the level of significance within the range of (0.0042–0.05). The Mann-Whitney U test was also used to conduct comparisons in homeless participants grouped by their BISQ scores (negative/low versus moderate/high). Similarly, the same test was used to compare FTT and GPT test performances between (1) homeless males and control males and (2) homeless females and control females.

The primary outcome of lifetime exposure to TBI was obtained by grouping participants with negative/low BISQ score as “no TBI” and moderate/high BISQ scores as “TBI.” Fifteen participants had no BISQ scores; therefore, data from their GIQ were used to define their TBI status. Kruskal-Wallis rank-sum test, and if necessary post hoc analysis using Dunn test, was performed for homeless individuals with TBI, homeless individuals without TBI, and controls. A leave-one-out cross-validation based on random forest classifier^35^ (chosen for its ability to work with small sample sizes and feature spaces of high dimensionality^36^) was conducted to predict the TBI exposure of all participants using the combination of MRI, NP test results, and AUDIT and DAST scores and each of MRI, NP, and AUDIT and DAST scores. These four data variations were also used to predict homelessness status in all participants and TBI exposure of homeless participants using random forest classifier.

## Results

### Demographics and questionnaires

Table 1 displays demographic characteristics of both the homeless and control groups, matched on age, sex, and post-secondary education. Significant differences in employment, income, and AUDIT scores (*p <* 0.001**) were observed. Homeless participants were more likely to have low and moderate/high BISQ scores, whereas controls had more negative BISQ scores (Table 1).

**Table 1.** Demographic Characteristics of the Homeless and Control Groups

Demographics	Homeless (*n* = 25)	Control (*n* = 26)
TBI^a^		
Yes	9	0
No	16	26
BISQ		
Negative	5	10
Low	11	3
Moderate	7	0
High	0	0
Incomplete/Declined to answer	2	13
Sex^b^		
Male	13	12
Female	12	14
Age		
Mean ± SD		51.6 ± 10.5	47.65 ± 16.3
Range		29–67	23–77
Income			
<$10, 000	13	1	
>$10, 000	8	21	
Declined to answer	4	4	
Education			
High school or less	10	3	
Post-secondary education	15	23	
Employment			
Employed	3	17	
Social assistance	18	8	
Declined to answer	4	1	
GIQ			
AUDIT (mean ± SD)		16 ± 12.60	3.19 ± 3.83
DAST (mean ± SD)		5.12 ± 5.52	2.38 ± 1.27

^a^ TBI status was determined by either BISQ or GIQ (if participant declined to answer the BISQ).

^b^ A chi-squared test showed that gender distribution did not vary significantly between homeless and controls.

AUDIT, Alcohol Use Disorders Identification Test; BISQ, Brain Injury Screening Questionnaire; DAST, Drug Abuse Screening Test; GIQ, General Information Questionnaire; TBI, traumatic brain injury; SD, standard deviation.

### NP tests: FTT and GPT

**FIG. 1.**
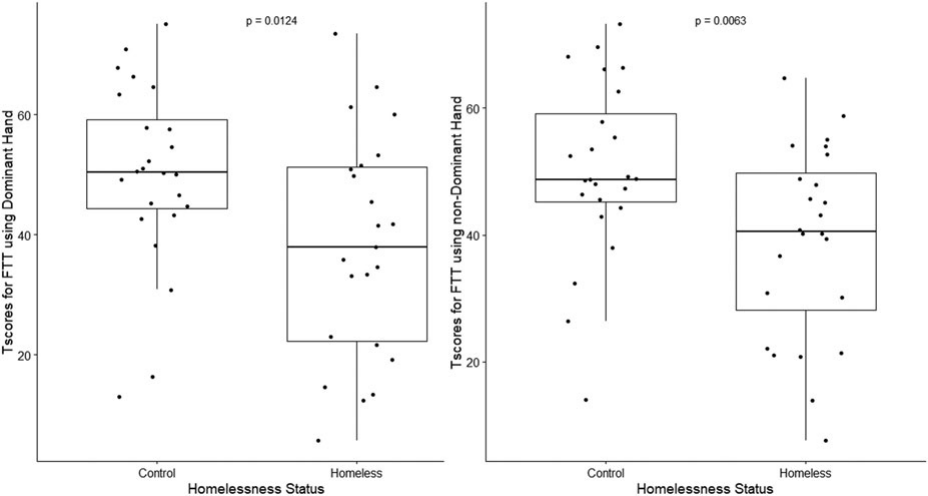
Boxplots for T-scores from the FTT results for homeless participants and controls using the dominant (left image) and non-dominant (right image) hands. The *p*-values are from one-sided Mann Whitney U test between homeless participants and controls. FTT, Finger Tapping Test.

The homeless group had lower T-scores compared with the controls with both dominant (*p =* 0.0124) and non-dominant (*p =* 0.0063) hands (Fig. 1). Moreover, both homeless males and females performed worse than their control counterparts on the FTT using their non-dominant hand (Table 2).

**Table 2.** Comparison of Mean T-scores of FTT and GPT Performance between Homeless Males/Females versus Control Males/Females

	Males			Females		
	Homeless	Controls	P-value	Homeless	Controls	P-value
FTT Dominant hand	38.62	48.00	0.0913	37.61	52.14	0.0259
FTT Non-dominant hand	37.75	51.05	0.0235	40.37	49.43	0.0740
GPT Dominant hand	130.63	61.37	0.0076	126.24	60.76	0.0055
GPT Non-dominant hand	104.79	58.52	0.0025^**^	123.07	46.21	0.0004^**^

^**^ Data that achieved less than a 0.0042 level of significance.

FTT, Finger Tapping Test; GPT, Grooved Pegboard Test.

In addition, both homeless and control groups performed with a lower T-score when using their dominant hand (mean T-score for homeless: 38.18, 95% CI: 30.17-46.19, mean T-score for controls: 50.07, 95% CI: 43.62-56.51) compared with their non-dominant hand (mean T-score for homeless: 38.95, 95% CI: 32.58-45.32, mean T-score for controls: 50.24, 95% CI: 44.34-56.13). Homeless participants with a moderate/high BISQ score had lower T-score values for the number of finger taps than controls when using their dominant hand (*p =* 0.0096). In addition, homeless participants with both low/negative BISQ (*p =* 0.0093) and moderate/high BISQ (*p =* 0.0148) tapped less than controls when using their non-dominant hand.

**FIG. 2.**
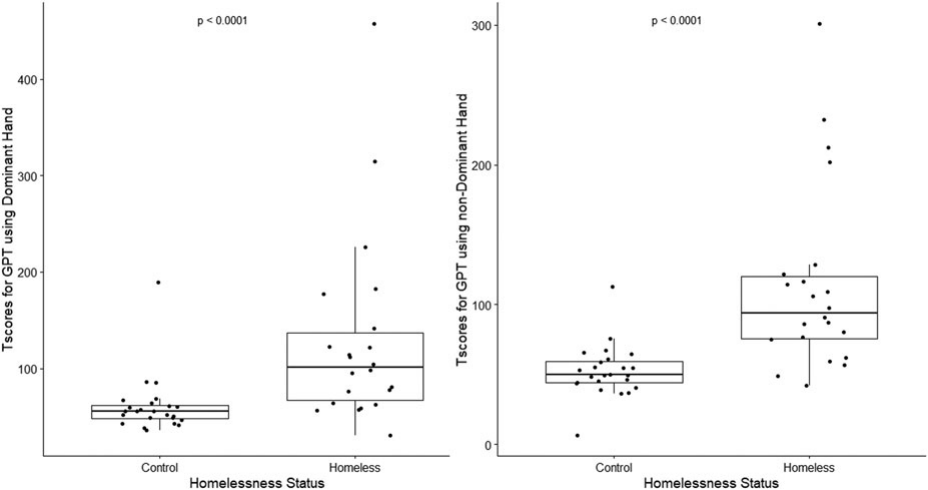
Boxplots for T-scores from the GPT results for homeless participants and controls using the dominant (left image) and non-dominant (right image) hands. The *p*-values are from one-sided Mann Whitney U test between homeless participants and controls. GPT, Grooved Pegboard Test.

The homeless group recorded significantly higher T-scores for time to complete the GPT using either their dominant (*p <* 0.0001**) or non-dominant (*p <* 0.0001**) hand. In addition, both homeless and control groups performed with higher T-scores when using their dominant hand (mean T-score for homeless: 128.64, 95% CI: 85.12-172.14, mean T-score for controls: 61.07, 95% CI: 48.34-73.79) compared with their non-dominant hand (mean T-score for homeless: 113.92, 95% CI 84.62-143.21, mean T-score for controls: 52.37, 95% CI: 44.45-60.27) (Fig. 2). In addition, both homeless males and females performed significantly worse than their control counterparts on the GPT using their non-dominant hand (Table 2).

Performance time of homeless individuals with BISQ scores of 0 and 1 was much longer (achieved higher T-scores) than that from controls using both dominant (*p =* 0.0003**) and non-dominant hands (*p =* 0.0003**). The performance time of homeless individuals with BISQ scores of 2 and 3 was also much longer (achieved higher T-scores) compared with that of controls using dominant (*p =* 0.0020**) and non-dominant hands (*p =* 0.0002**).

Kruskal-Wallis rank-sum test revealed that FTT results using non-dominant hands and GPT using both dominant and non-dominant hands varied significantly (*p <* 0.05) among the three groups. Post hoc analyses found that pairwise differences were present between homeless participants without TBI and controls for the FTT using non-dominant hands (*p =* 0.0071) and the GPT with dominant (*p =* 0.0002**) and non-dominant hands (*p =* 0.0002**). Homeless participants with TBI and controls significantly differed in their performance on the GPT with dominant (*p =* 0.0027**) and non-dominant hands (*p =* 0.0001**).

### MRI brain volumes

Among the MRI subsegmented regions of the brain considered for this study, both thalamus and brainstem volume estimates expressed over the estimated total ICV were less in homeless participants compared with controls (*p <* 0.001**). Reduced proportion (normalized volumes with respect to estimated total ICV) of volumes were noted for the accumbens, thalamus, ventral diencephalon, and brainstem (*p <* 0.001**) in TBI compared with non-TBI homeless participants.

### Combined effect of NP tests, AUDIT, DAST, and MRI brain volumes on predicting TBI

**FIG. 3.**
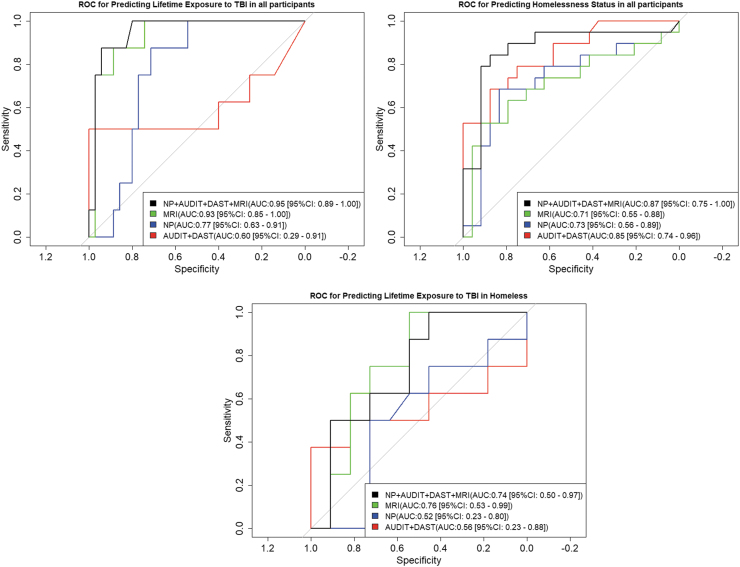
Receiver Operating Characteristic (ROC) curves using the probabilities predicted for lifetime exposure to TBI (left image, top row) and homelessness status (right image, top row) for all participants, and lifetime exposure to TBI in homeless participants only (bottom row) by random forest classifiers based on different combinations of variables. AUC, area under the ROC curve; AUDIT, Alcohol Use Disorders Identification Test; CI, confidence interval; DAST, Drug Abuse Screening Test; TBI, traumatic brain injury; NP, neuropsychological; MRI, magnetic resonance imaging.

Participants with missing FTT and GPT data were excluded to only include participants with complete data. This resulted in 8 participants with TBI exposure and 35 without. Our random forest classifier using MRI data, NP results, AUDIT and DAST scores (used with default hyper parameters in the R randomForest package) under the leave-one-out classification scheme was able to discriminate between participants based on their TBI status (determined via BISQ scores or the GIQ) with an area under the receiver operating characteristic (ROC) curve (AUC) of 0.95 (95% CI: 0.89-1.00). Using Delong's test, we found that the AUC using MRI, NP tests, and questionnaire data (AUDIT and DAST) was better (*p <* 0.05) than random forest trained with NP data (AUC: 0.77, 95% CI: 0.63-0.91) or questionnaire data (AUDIT and DAST) only (AUC: 0.60, 95% CI: 0.29-0.91). However, the AUC using MRI data, NP test results, and AUDIT and DAST scores was comparable to the predictive ability obtained using the MRI extracted brain region volumes only (AUC: 0.93, 95% CI: 0.85-1.00). The ROCs for these tests are shown in Figure 3. When TBI was predicted in homeless participants only (*n* = 8 with TBI, *n* = 11 without TBI), AUC decreased and the classifier using MRI (AUC: 0.76, 95% CI: 0.53-0.99) only was found to perform better than the other three.

When homelessness status was predicted in all participants (*n* = 19 homeless, *n* = 24 controls), the highest AUC (AUC: 0.87, 95% CI: 0.75-1.00) was obtained through the combination of MRI data, NP results, and AUDIT and DAST scores. Moreover, AUDIT and DAST scores (AUC: 0.85, 95% CI: 0.74-0.96) performed better than that of NP (AUC: 0.73, 95% CI: 0.56-0.89) and MRI (AUC: 0.71, 95% CI: 0.55-0.88) individually for predicting homelessness.

**FIG. 4.**
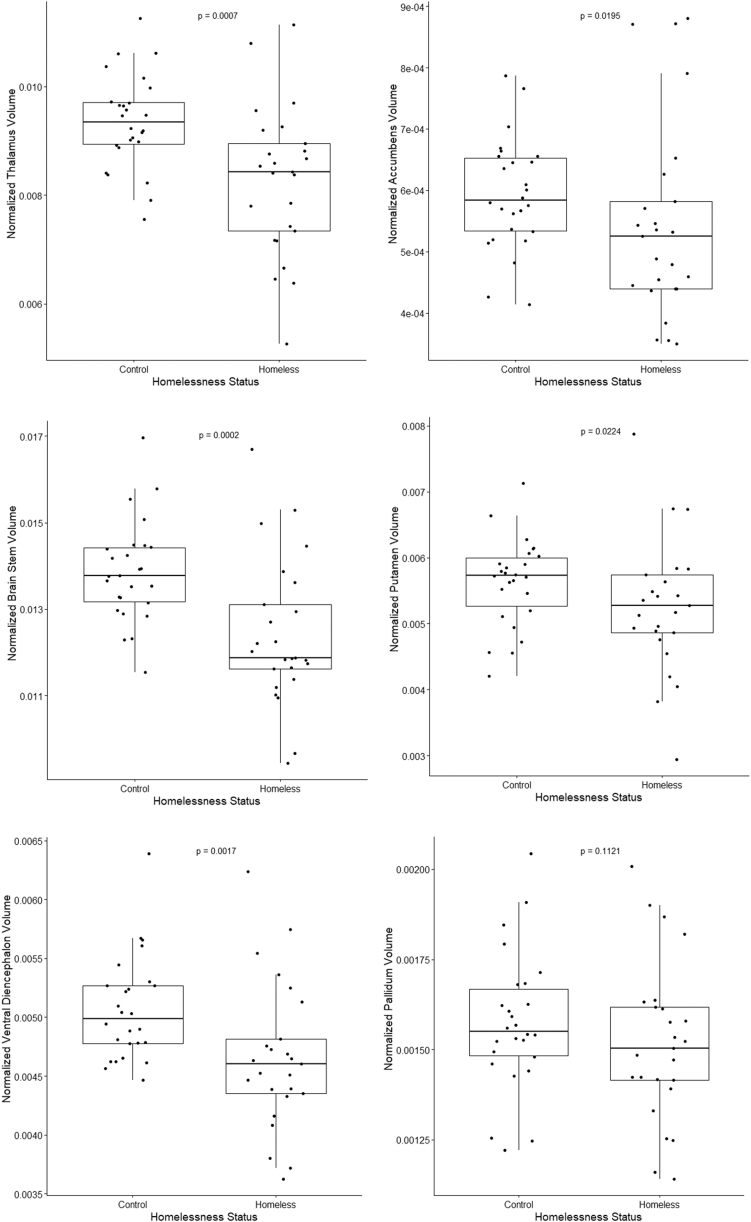
Boxplots for normalized volumes of various brain regions for homeless participants and controls. The *p*-values are from two-sided Mann Whitney U test between homeless participants and controls.

**FIG. 5.**
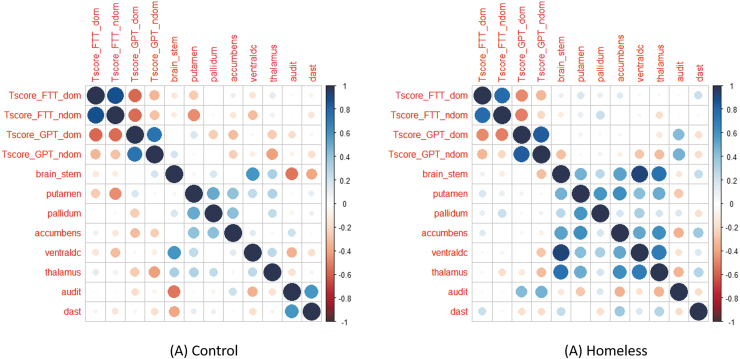
Pearson's correlation between the variables related to the FTT, GPT, brain volumes, AUDIT, and DAST scores for controls and homeless participants. The variables listed in the figures are defined as follows: Tscores_FTT_dom: T-scores for FTT using dominant hand; Tscores_FTT_ndom: T-scores for FTT using non-dominant hand; Tscores_GPT_dom: T-scores for GPT using dominant hand; Tscores_GPT_ndom: T-scores for GPT using non-dominant hand; brain_stem: normalized brainstem volume; putamen: normalized putamen volume; pallidum: normalized pallidum volume; accumbens: normalized accumbens volume; ventraldc: normalized ventraldc volume; thalamus: normalized thalamus volume; audit: AUDIT scores; dast: DAST scores. The color codes correspond to the values indicated in the legend. The radii of the circles increase with the increase of the absolute value of the correlation. AUDIT, Alcohol Use Disorders Identification Test; DAST, Drug Abuse Screening Test; FTT, Finger Tapping Test; GPT, Grooved Pegboard Test.

Finally, we were able to demonstrate differences between the homeless and control groups in the correlations between subsegmented brain volumes and cognitive testing, as well as correlations between subsegmented brain volumes and AUDIT/DAST results (Figs. 4 and 5). High pairwise correlation between subsegmented brain volumes was noted in the homeless group compared with the controls. Higher negative correlations were noted between each of the brainstem and ventral diencephalon volumes and DAST and AUDIT score for controls compared to homeless participants. This is illustrated in Figure 5 by the significantly higher subsegmented brain volumes seen in the controls versus homeless participants.

## Discussion

Our study is unique in that it studied a cohort of homeless individuals with and without TBI using detailed volumetric MRI. Detailed brain volume analyses in our study showed significant differences in the volumes of several brain regions, notably the thalamus and brainstem, in the homeless participants compared with controls (*p <* 0.001**). Further, despite a small sample size, we showed reduced normalized volumes (with respect to estimated total ICV) in the nucleus accumbens, thalamus, ventral diencephalon, and brainstem (*p <* 0.001**) in TBI homeless participants compared with non-TBI homeless participants. Other studies focusing on athletes with concussion have shown that former athletes with at least one grade 3 concussion had lower mean hippocampal volumes bilaterally compared with control participants^37^ and smaller amygdala volumes after adjusting for multiple comparisons.^38^ Our findings are more similar to those seen in patients who have sustained diffuse axonal injury^39^ and provide evidence that caregivers of homeless patients, particularly of homeless patients with a history of TBI, would benefit from policies, strategies, and interventions used with TBI patients, and should be educated about the deficits expected in the homeless.

We also identified a series of significant cognitive deficits and MRI volume differences in homeless persons with a history of TBI, compared with a group of homeless persons without TBI and with controls. Notably, homeless participants with a history of TBI scored particularly poorly for non-dominant hand performance and on GPT times more so than the FTT suggesting that the combination of TBI and homelessness affected the GPT to a greater extent. Although the diverse cognitive sequelae of TBI that consist of deficits in working memory, attention, information processing, and other executive functions that may linger for months, even years, following injury are well documented, studies that document these findings in homeless individuals are scarce, and so highlight the relevance of our findings.

The homeless group showed less of a negative correlation between putamen volumes and FTT T-scores in both dominant and non-dominant hands when compared with the control group. This suggests that sizes of putamen MRI volumes highly varied in the homeless population, which could be attributed to the prevalence of TBI. These results are further supported by the known relationship between functional MRI activation of the putamen with finger tapping and its volume loss when associated with TBIs.^40,41^ Homelessness in our sample was associated with brain volume reduction and worse NP test performance on a consistent basis. The GPT was associated with a greater number of brain volume correlates than the FTT, which is consistent with its greater task complexity, as it requires a wider span of executive functions.^11,14^

In addition, the results confirmed relatively high rates of alcohol and drug use in our homeless cohort with or without TBI. Although we did not have the power to show significant differences in the TBI homeless group, there were trends supporting more of these associated behaviors in our TBI homeless group. Our results also showed that the homeless population demonstrated a stronger positive correlation for both dominant and non-dominant hands between GPT and AUDIT T-scores in contrast to the control population, who did not demonstrate any strong correlation between these measures. This may simply reflect the concept that persons with alcohol use disorder score worse on measures reflecting visual-spatial motor skills^42^; however, our data were significant for non-dominant hand performance, suggesting that there may be more severe deficits associated with the combination of TBI and homelessness.

We were able to demonstrate differences between the homeless and non-homeless groups in the correlations between subsegmented brain volumes and cognitive testing, as well as correlations between subsegmented brain volumes and AUDIT/DAST results. Several studies show that alcohol abuse is a risk factor of volumetric changes in the brain.^43–45^ In athletes with repeated concussion, smaller subcortical volumes have only been found in rugby players with histories of combined alcohol use and repetitive neurotrauma and not in those without significant alcohol use,^46^ suggesting either a primacy to the alcohol use or a particularly damaging combination of alcohol and TBI. Our results suggest that homelessness may add a further susceptibility to those already vulnerable to TBI and alcohol use.

Our combined effect of NP tests, AUDIT, DAST, and MRI brain volumes on predicting TBI demonstrated superiority compared with predicting TBI with either of these measures alone. However, it is important to note that the difference between predicting TBI with our predictive model compared with MRI alone was not significant. As such, it is possible that MRI alone allows for effective and objective prediction of TBI. Therefore, if we had applied more than two of the possible NP tests or recruited additional participants, the combinatory power of our predictive values may have been further strengthened. It should be noted that participants with TBI were all homeless and those without TBI were either homeless or controls. The better discriminatory performance of the TBI classifier (using three of the four data variations) indicates that possible effects of TBI were more prominent in comparison with the homelessness status in the participants with TBI using the combined, NP, and MRI data. Within the homeless participants, MRI predicted presence of TBI with the best AUC. This further indicates that effects of TBI are likely to be more prominent in MRI, compared with that of homelessness. In contrast, AUDIT and DAST scores together discriminated homelessness status better than TBI.

Previous studies have suggested a causal link between TBI and homelessness with reports of 70–90% of subjects experiencing TBI prior to the onset of homelessness.^3,5^ However, our study design is only able to draw an association between TBI and homelessness, not a causal relationship. Our findings of cognitive deficits may indicate that homelessness, combined with the cognitive impairments associated with TBI, increases an individual's vulnerability to subsequent injury and TBI, creating a repetitive cycle of TBI and homelessness. This is a reasonable hypothesis given that prior research shows that regardless of socioeconomic or cultural background, impairment from TBI worsens quality of life, increases risk of anxiety, depression, and suicide for up to 10 years following injury,^47,48^ and is associated with lower rates of employment and a decrease in yearly income compared with pre-injury.^49^ This underscores the importance of elucidating specific patterns of cognitive deficits to those caring for homeless individuals with TBI and in the rehabilitation of those with TBI to prevent subsequent homelessness.

### Limitations

Despite the small sample sizes of our groups, we corrected for multiple tests of significance and still found significant differences between the homeless individuals and the controls and between the homeless participants with and without TBI. Our data are subject to self-report, recall, and desirability bias in the responses to the AUDIT, DAST, and BISQ particularly because both TBI and alcohol may have effects on memory. However, MRI volumes would not be subject to such bias. There is also the inherent possibility of error introduced by interpretation of images based on MRI acquisition protocols, automatic pre-processing and segmentation routines applied on the MRI acquired volumes. The classification performance was evaluated under a leave-one-out cross-validation scheme, which restricts the understanding of the generalizability of our results. Tests on external/hold-out sets are necessary to validate our findings further. Finally, our study was cross-sectional so can only document associations and cannot infer causality.

## Conclusion

Volumetric changes were seen in homeless participants regardless of TBI status, which resemble findings seen in patients with diffuse axonal injury. Homeless participants performed more poorly than controls on NP tests, with homeless participants with TBI performing more poorly than those without TBI. NP tests, AUDIT scores, DAST scores, and subsegmental neuroanatomical volumes from MRI predicted the lifetime exposure of TBI in participants with a better AUC than their homelessness status.

Policies, strategies, and interventions for people with TBI and people who are homeless must take into account the overlapping susceptibilities created by TBI, homelessness, and alcohol and drug use. Caregivers of TBI patients and those caring for homeless clients should seek to increasingly develop strategies, policies, and interventions that address the combined effects of TBI and homelessness to optimize outcomes. Future work with larger sample sizes and longitudinal designs may help to disentangle some of the confounding factors and provide further insights into causality and the relative roles of factors perpetuating TBI and homelessness.

## Abbreviations Used

AUC: area under the curve
AUDIT: Alcohol Use Disorders Identification Test
BISQ: Brain Injury Screening Questionnaire
CI: confidence interval
DAST: Drug Abuse Screening Test
DTI: diffusion tensor imaging
FLAIR: fluid-attenuated inversion recovery
FOV: field of view
FTT: Finger Tapping Test
GIQ: General Information Questionnaire
GPT: Grooved Pegboard Test
ICV: intracranial volume
MRI: magnetic resonance imaging
NP: neuropsychological
ROC: receiver operating characteristic
ROI: region of interest
TBI: traumatic brain injury
TE: echo time
TR: repetition time
